# The Overexpression of a Transcription Factor Gene *VbWRKY32* Enhances the Cold Tolerance in *Verbena bonariensis*

**DOI:** 10.3389/fpls.2019.01746

**Published:** 2020-01-29

**Authors:** Meng-qi Wang, Qiu-xiang Huang, Ping Lin, Qin-han Zeng, Yan Li, Qing-lin Liu, Lei Zhang, Yuan-zhi Pan, Bei-bei Jiang, Fan Zhang

**Affiliations:** ^1^Department of Ornamental Horticulture, Sichuan Agricultural University, Chengdu, China; ^2^The Key Laboratory of Plant Resources Conservation and Germplasm Innovation in Mountainous Region (Ministry of Education), Institute of Agro-Bioengineering and College of Life Sciences, Guizhou University, Guiyang, China

**Keywords:** *Verbena bonariensis*, cold stress, *VbWRKY32*, antioxidant enzymes, osmotic adjustment, down-stream genes

## Abstract

Cold stress poses a serious threat to the survival and bloom of *Verbena bonariensis*. The enhancement of the cold tolerance of *V. bonariensis* is the central concern of our research. The WRKY transcription factor (TF) family was paid great attention to in the field of abiotic stress. The *VbWRKY32* gene was obtained from *V. bonariensis*. The *VbWRKY32* predicted protein contained two typical WRKY domains and two C2H2 zinc-finger motifs. Under cold stress, *VbWRKY32* in leaves was more greatly induced than that in stems and roots. The overexpression (OE) in *V. bonariensis* increased cold tolerance compared with wild-type (WT). Under cold stress, the OE lines possessed showed greater recovery after cold-treatment restoration ratios, proline content, soluble sugar content, and activities of antioxidant enzymes than WT; the relative electrolyte conductivity (EL), the accumulation of malondialdehyde (MDA), hydrogen peroxide (H_2_O_2_), and superoxide anion (O_2_^−^) are lower in OE lines than that in WT. In addition, a series of cold-response genes of OE lines were compared with WT. The results revealed that *VbWRKY32* worked as a positive regulator by up-regulating transcription levels of cold-responsive genes. The genes above can contribute to the elevation of antioxidant activities, maintain the membrane stability, and raise osmotic regulation ability, leading to the enhancement of the survival capacity under cold stress. According to this work, *VbWRKY32* could serve as an essential gene to confer enhanced cold tolerance in plants.

## Introduction

Plants are often subjected to various stresses which affects their growth and development. Some genes, including WRKY transcription factors (TFs), are induced to help plants adapt to stresses by changing physiology and morphology. WRKY TFs were a valuable family which resisted abiotic stress, such as cold, NaCl, drought, salicylic acid, ethylene, abscisic acid, methyl jasmonate, and hydrogen peroxide (H_2_O_2_) (Wang et al., 2013; Zhou et al., 2015; Xiu et al., 2016). The WRKY family comprised one or two DNA-binding domains consisted of 60 amino acid regions and the highly conservative sequence WRKYGQK at its N-terminus and a zinc-finger motif (Cx_4−5_ Cx_22−23_HxH or Cx_7_Cx_23_HxC) at C-terminus (Eulgem et al., 2000; Rushton et al., 2010). In terms of the structure of WRKY protein, it could be divided into three groups: WRKY group I (contained two WRKY domains with Cx_4−5_Cx_22−23_HxH zinc-finger motifs), group II (one WRKY domain with a Cx_4−5_Cx_22−23_HxH motif), and group III (one WRKY domain with a C-X7-C-X23-H-X-C motif).

Several WRKY-overexpressed plants had successfully enhanced the resistance to various abiotic stresses. For example, overexpression of *IIWRKY2* (WRKY group II) gene elevated salt tolerance in overexpression (OE) lines of *Iris lactea* var. *chinensis* (Tang et al., 2018). *OsWRKY11* (WRKY group II) functioned as a positive regulator in tolerance to heat and salt stress of the transgenic rice seedlings (Wu et al., 2009). *OsWRKY45* promoted the resistance to disease and drought in *Arabidopsis* (Qiu and Yu, 2009). Compared with wild type (WT), the *CsWRKY46* (WRKY group II)-overexpressed cucumber *via* regulating a series of regulated cold-responsive genes raised cold tolerance (Ying et al., 2016). *OsWRKY71* (WRKY group II) has a positive function in cold tolerance by regulating downstream target genes in rice (Kim et al., 2016). Overexpressed *FcWRKY70* (WRKY group III) in tobacco and lemon conferred enhanced tolerance to drought stresses (Gong et al., 2015). Overexpression of *GhWRKY25* (WRKY group I) in *Nicotiana benthamiana* enhanced plant tolerance to salt stress (Liu et al., 2016). TFs are significant for cold signaling and tolerance by modulating the expression of related functional genes (Nair et al., 2009). WRKY are vital regulators in certain development processes. Overexpressed *OsWRKY30* (WRKY group I) enhanced rice resistance to disease by the salicylic acid (SA) signaling pathway (Ryu et al., 2006). The *WRKY34* (WRKY group I) TF negatively regulated cold sensitivity of mature *Arabidopsis* pollen and might be involved in the C-repeat binding factor (CBF) signal cascade in mature pollen (Zou et al., 2010). However, WRKY group I and III members have been rarely reported compared with group II members, in particular in response to stress. Therefore, combined with the analysis of transcriptomic data, WRKY group I will be the focus of our attention.

*Verbena bonariensis* is a perennial herb native to South America (Brazil, Argentina, etc.). With its high ornamental value and supreme drought resistance, *V. bonariensis* is widely used in flower border and sightseeing farms. However, *V. bonariensis* owns inferior resistance of low temperature, which causes damage during flowering and impacts yield. When below 0°C or worse, chilling injury would result in destruction or death in production. In China, the studies on *V. bonariensis* are now mainly focused on seedling breeding, garden application, and salt tolerance. The low temperature molecular research in *V. bonariensis* has not been reported. Thus, it is urgent to determine and improve the cold resistance in *V. bonariensis*. The transcriptomic data of *V. bonariensis* in cold stress displayed that WRKY TFs worked as a vital role in helping plants cope with low temperatures stress.

In this study, we isolated and cloned *VbWRKY32* gene from *V. bonariensis*. The analysis of *VbWRKY32* expression responding to cold stress was elaborated from multiple angles. Overexpressed *VbWRKY32* in *V. bonariensis* elevated tolerance of cold stress, compared with WT. The statistics indicated that *VbWRKY32* could serve as a new candidate gene to accomplish the cultivation of cold-tolerant plants and contribute more detailed information to WRKY family.

## Materials and Methods

### Plant Materials

*V. bonariensis*, c.v. *Finesse* was selected as research materials. The *VbWRKY32* gene was cloned from tissue-cultured seedlings which were grown on Murashige and Skoog (MS) medium. *V. bonariensis* were cultivated in the greenhouse of Sichuan Agricultural University. The two OE lines (OE-1 and OE-5) and WT of *V. bonariensis* were tested in our study.

### Clone of the *VbWRKY32*

Total RNA extraction of tissue-cultured *V. bonariensis* leaves was achieved by TRIzol Reagent (MyLab, Beijing, China). The synthesis of complementary DNA (cDNA) was accomplished by Takara^®^ PrimeScript™ RT Reagent Kit with gDNA Eraser (Perfect Real Time) (TAKARABIOIN, Beijing, China). The full-length cDNA of *VbWRKY32* was acquired by PCR. The *VbWRKY32* gene was cloned using specific primers (F: CGTAAAGAAAAGAAAAAGCTTTTAT; R: CGCTACCACTACAATCAACCTATAT). PCR was performed in 25-μl reaction volume containing 1 μl cDNA, 0.5 μl each primer, 10.5 μl double-distilled H_2_O (ddH_2_O), and 12.4 μl Taq Mix. The program employed was 30 cycles of 94°C for 30 s, 56°C for 30 s, and 72°C for 1 min; finally stored at 4°C. The cloned sequence was inserted into pCAMBIA 2300 with the control of cauliflower mosaic virus (CaMV) 35S promoter to obtain P*_VbWRKY32_*. The enzyme restriction sites were *BamH I* and *Kpn I*.

### Phylogenetic and Conserved Domain Analysis of *VbWRKY32*

Phylogenetic analysis was conducted by MEGA version 4.0 using 34 *VbWRKY32* homologs selected from multiple sequence alignment. Sequence alignment was performed using DNAMAN.

### Transformation

The regenerated explants were selected from upper middle leaves of *V. bonariensis*. The P*_VbWRKY32_* construct was introduced into *Agrobacterium tumefaciens* strain GV3101 and transformed into leaves of *V. bonariensis*. The mediums used for transformation were showed in **Table 1**. The explants were cultured in the M1 medium for 2 days at 35°C and under the 16/8 h light/dark photoperiod with 3,000 lx intensity of illumination. The samples were infected with bacterial solution of *A. tumefaciens* (OD_600_ = 0.4) for 10 min and placed on the M1 medium. After 2-day dark culture, materials were transferred to the M2 medium in culture bottles. These explants were transferred to fresh M2 medium every 2 weeks to maintain appropriate selective pressure and at the same time to prevent the growth of *Agrobacteria*. After 35 days, the adventitious buds were cut off, transplanting to M3 medium. After rooting and elongation, the plantlets were identified by PCR and adapted to growth in the soil in the soil. The DNA secure Plant Kit (TIANGEN^®^DNAsecure) was used for the PCR procedure for the transgenic identification.

**Table 1:** The mediums in transformation process.

Serial no.	Name of medium	Composition of medium
**M1**	Pre-culture medium	4.4 g·L^−1^ MS+1.0 mg·L^−1^ 6-BA+0.1 mg·L^−1^ NAA+6.5 g·L^−1^ agar+30 g·L^−1^ sucrose
**M1**	Co-culture medium	4.4 g·L^−1^ MS+1.0 mg·L^−1^ 6-BA+0.1 mg·L^−1^ NAA+6.5 g·L^−1^ agar+30 g·L^−1^ sucrose
**M2**	Selective medium	4.4 g·L^−1^ MS+1.0 mg·L^−1^ 6-BA+0.1 mg·L^−1^ NAA+450 mg·L^−1^ CARB+6.5 g·L^−1^ agar+30 g·L^−1^ sucrose+1.0 mg·L^−1^ kana
**M3**	Rooting medium	4.4 g·L^−1^ MS+6.5 g·L^−1^ agar+30 g·L^−1^ sucrose

### Cold Treatment of Overexpression and Wild-Type Plants

Three-month old seedlings of *V. bonariensis* were transferred from the greenhouse (16 h photoperiod, 28°C/20°C day/night temperature) to the thermoregulating incubator. The seedlings were treated at the following temperature (this cooling process was continuous.): 4°C for 24 h (T2), followed by −4°C for 4 h (T3) and 6 h (T4). The upper leaves were harvested at the time points of the control (T1), T2, T3, and T4 for physiological experiments and histochemical detection of ROS, which were frozen in liquid nitrogen instantly and stored at −80°C. Roots, stems, and leaves of the same untreated seedlings were collected for the analysis of tissue-specific expression.

### Analysis of the Level of Gene Expression

The materials were treated under following conditions: 4°C for 24 h, followed by −4°C for 4 h. The quantitative real-time polymerase chain reaction (qRT-PCR) was performed in the SsoFast EvaGreen Supermix (Bio-Rad, Hercules, CA, United States) and Bio-Rad CFX96TM detection system. The *Actin-11* gene served as a quantitative control to detect expression level of *VbWRKY32* and of nine cold-related genes, including *VbCor413im1*, *VbCor413pm2*, *VbPOD*, *VbCAT*, *VbSOD*, *VbAPX6*, *VbP5CS*, *VbAMY3*, and *VbBAM1*. The 20 μl qRT-PCR reaction mixture was incubated at 95°C for 30 s, followed by 40 cycles at 95°C for 15 s, at 60°C for 30 s, then by a final single melt cycle from 65 to 95°C. Each reaction was carried out for three biological repetitions. Relative expression levels were calculated by the 2^−ΔΔCT^ method. All correlative primers of qRT-PCR were exhibited in **Table 2**.

**Table 2:** Primers used to quantitative real-time PCR (qRT-PCR).

Gene name	Forward primers	Reverse primers
*VbWRKY32*	GGTTATGCGTAAAGAAAAGAAAAA	TCTGCAGATACAAATCTAAATCACC
*Actin-11*	TGCAATATAAATTTATATCTGGATG	TATCAGCAATACCAGGAAACATAGT
*VbCor413im1*	CTATCTCTGTCTCTTTCTGATTCGC	AAATTTCATTGTTAAAAGGGGG
*VbCor413pm2*	TAGAGCATCTGGTGGATTCAGA	GGATCAGCAAACAATATGAAGAC
*VbPOD*	GCATTGCATACATGAATACATAACA	TTGTGTCTCATAGTTTTCGACCA
*VbCAT*	ACTTTATTAACAAAATTCCAAAGCT	GGTCTTGAAAATTAGTGTGTCAAGA
*VbSOD*	GACATAAACCTTTTATTAAACGACA	GAAAAGAAGGTAAGAAAATGATTCA
*VbAPX6*	ACCTAATTACTAAACCACGTCACAC	AAAAGAGCTTCTCAAGACTACGTTC
*VbP5CS*	TCTTTGTTGTCTCTGAAAGTTCCTA	TTCTTGAACTTTCTCTAGCTGCTAC
*VbAMY3*	TACTTACAACAGACCCAGTCTTCC	CAGCAATAGAACTGCTTGTATTAAA
*VbBAM1*	ACTTGTATTTTCTCAAACTCTCCCT	ATGCTCAGCAGTCTAAAAGCAG

### Freezing Recovery Ratios

Three-month-old seedlings (WT, OE-1, and OE-5) were selected to detect freezing recovery ratios. The materials were treated under following conditions: 4°C for 24 h, followed by −4°C for 4 h (wilting symptoms began to appear), finally allowed to recover for 10 days at 28°C. The freezing recovery ratios were measured. It was recorded that less than 25% of withered leaves in seedlings were regarded as recovery.

### Determination of Physiological Indexes

Malondialdehyde (MDA) content and electrolyte conductivity (EL) were measured according to Lei et al. (2009) and Wang et al. (2017b), respectively. Activities of superoxide dismutase (SOD), peroxidase (POD), catalase (CAT), and ascorbate peroxidase (APX) were measured by the methods of Beauchamp and Fridovich (1971), Ranieri et al. (2000), and Zhang et al. (2011), respectively. The content of soluble protein, soluble sugar, and proline was measured following Wang et al. (2013) and Irigoyen et al. (2010).

### Histochemical Detection of Reactive Oxygen Species

The accumulation of H_2_O_2_ and superoxide anion (O_2_^−^) in leaves was detected by the method of histochemical staining using 3,3′-diaminobenzidine (DAB) and nitroblue tetrazolium (NBT), respectively (Wang et al., 2017a). Finally, the stained leaves were photographed. H_2_O_2_ and O_2_^−^ content were measured following An (2012) and Yamamoto et al. (2001).

### Statistical Analyses

All experiments were performed for three biological repeats. The data were analyzed *via* one-way analysis of variance using SPSS version 19.0, and statistically significant differences were calculated with p < 0.05 as the thresholds for significance.

## Results

### Isolation and Sequence Analyses of *VbWRKY32*

To obtain valuable candidate genes from *V. bonariensis* and to facilitate the molecular culture of cold-resistant varieties, next generation sequencing technique were applied to construct a cDNA library out of *V. bonariensis* leaves (Wang et al., 2019). The RNASEQ data it was deposited in National Center for Biotechnology Information (NCBI) (NCBI GEO series GSE112477, NR ID: XP_011083976.1). The *WRKY32* TF (Cluster-14918.129050) was selected from differentially expressed genes (DEGs) with functional annotations, and named *VbWRKY32*. Its log_2_ (fold change) was 5.45 and q value was less than 0.01.

**Figure 1:**
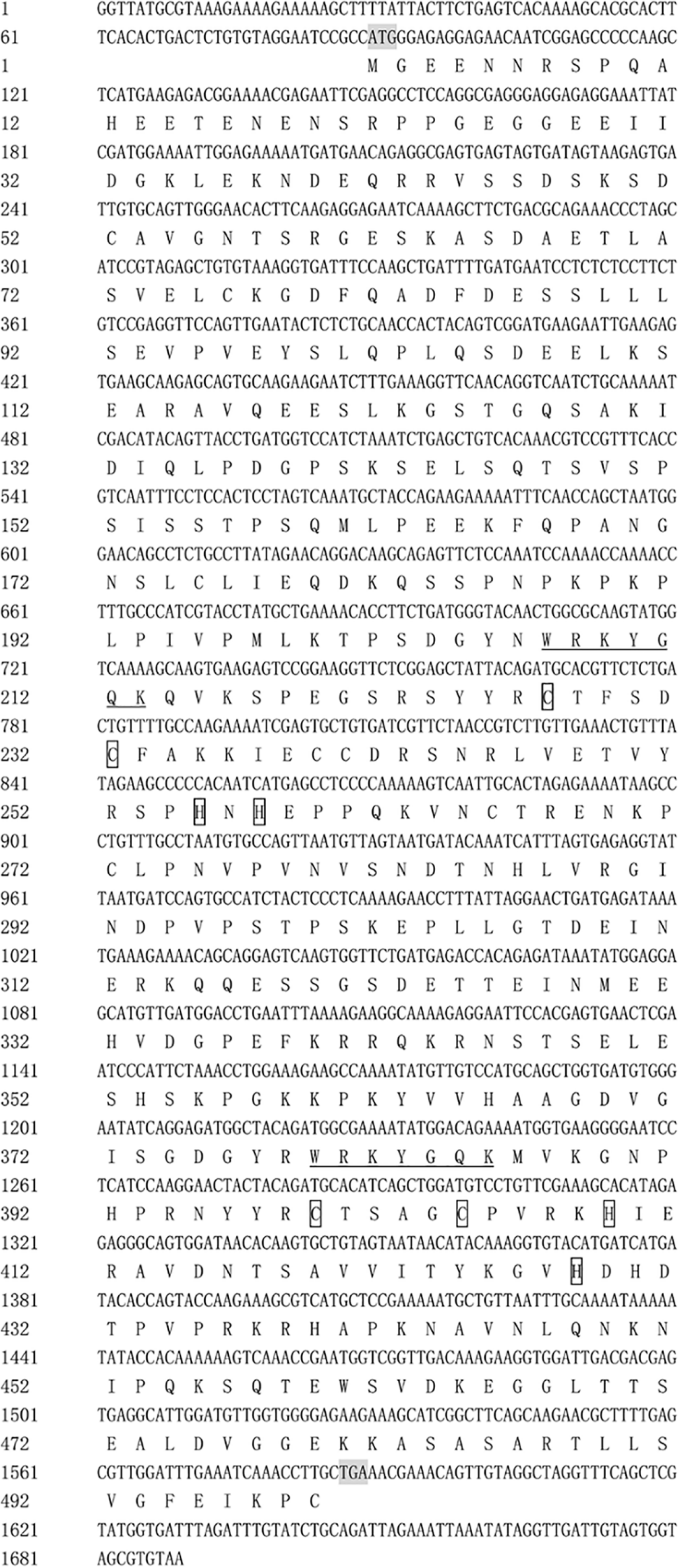
Nucleotide and deduced amino acid sequences of *VbWRKY32*. The WRKY domain is underlined. The two cysteines and two histidines in the zinc-finger motifs are framed.

**Figure 2:**
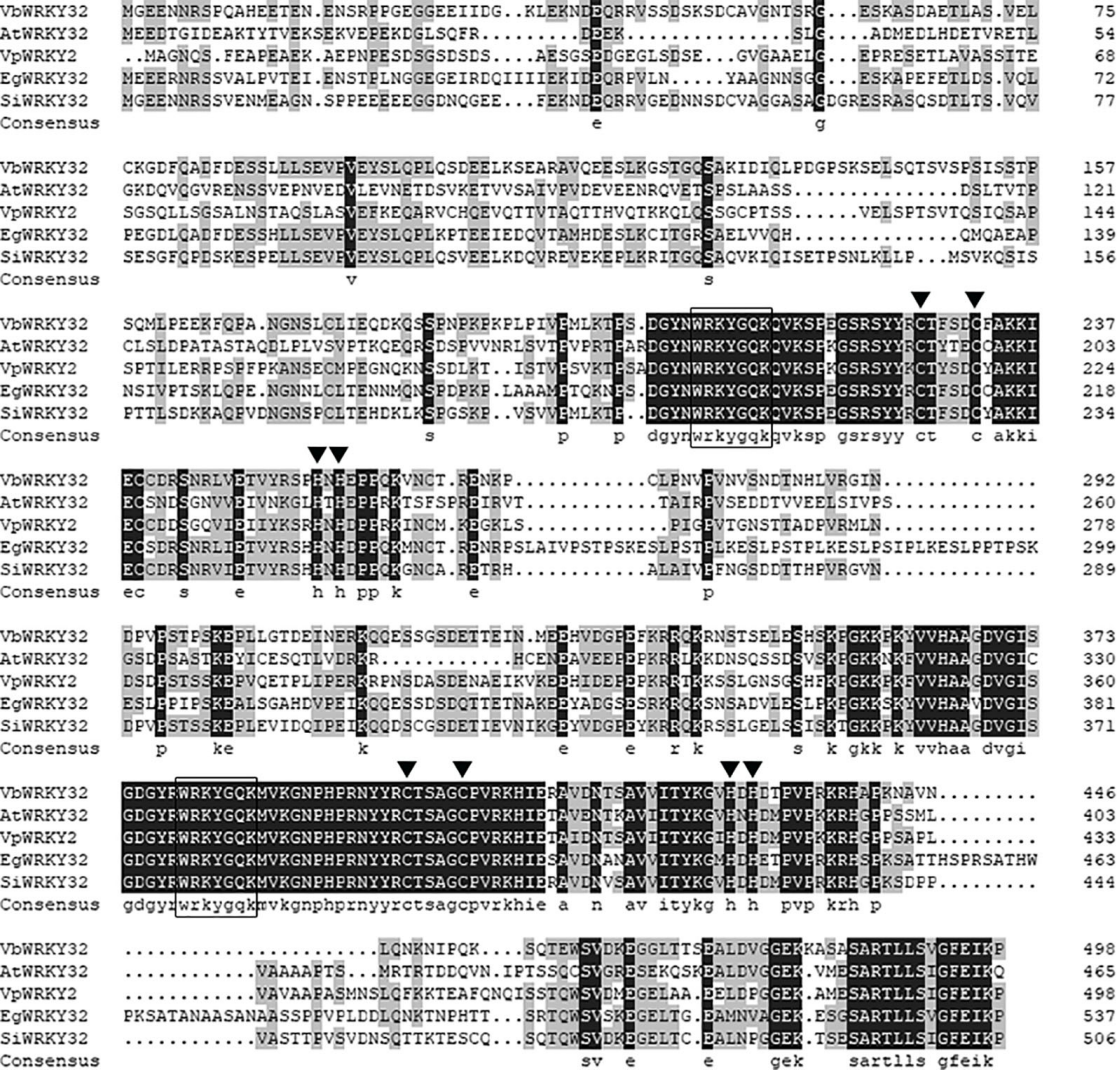
Comparison between the amino acid sequences deduced by *VbWRKY32* gene and the multisequences of WRKY protein from other plants. Identical amino acid residues are shaded in black boxes, others meet two identical residues in gray boxes. The completely conserved WRKYGQK amino acids are enclosed by oblong. The cysteine (C) and histidine (H) in zinc-finger motifs are pointed out by black trigon. *AtWRKY2* (NP_200438.1) from *Arabidopsis thaliana*; *VpWRKY2* (GU565706) from *Vitis pseudoreticulata*; *EgWRKY32* (XP_012841117.1) from *Erythranthe guttata*; *SiWRKY32* (XP_011083976.1) from *Sesamum indicum*.

**Figure 3:**
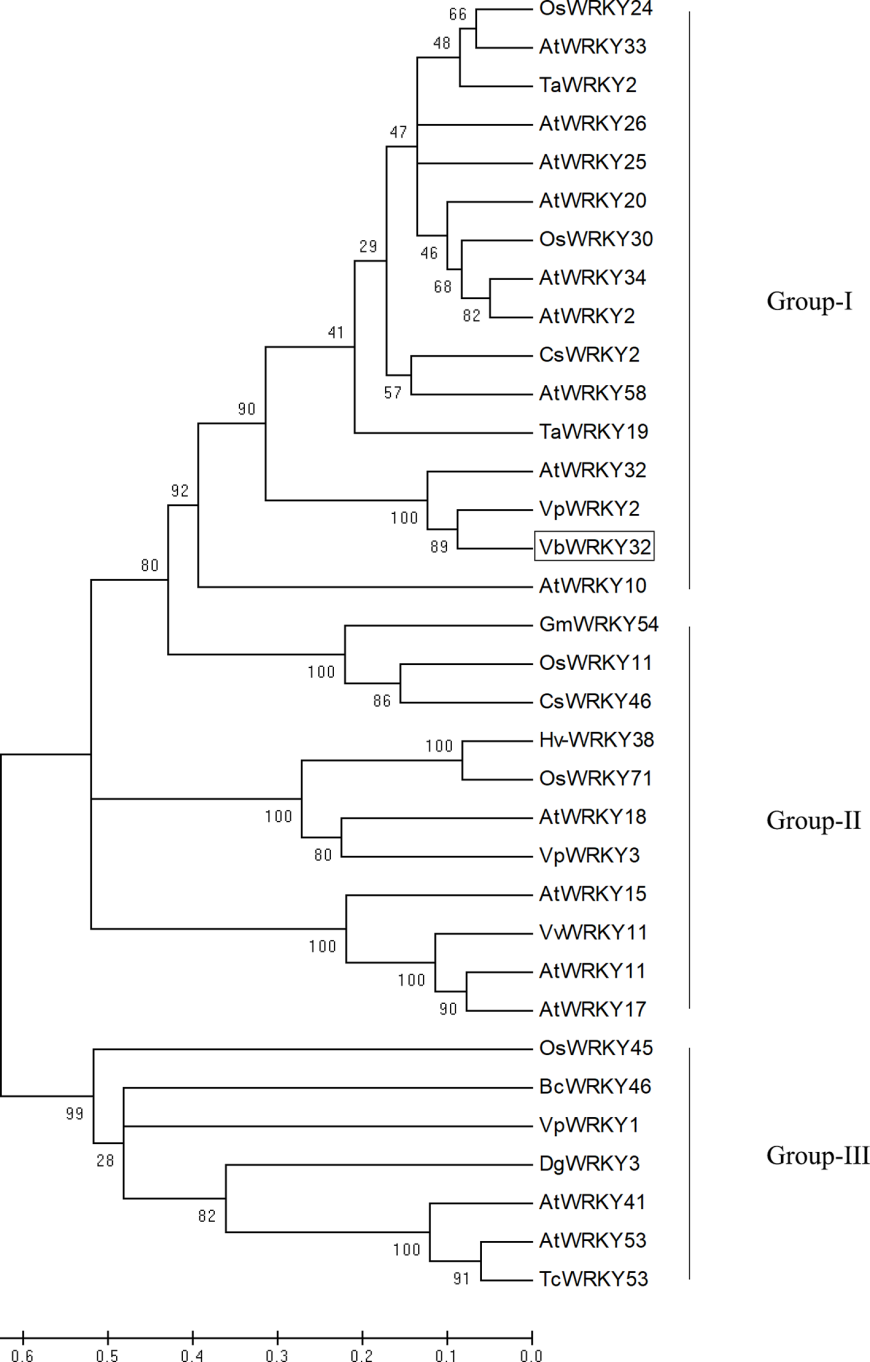
Phylogenetic tree analysis of *VbWRKY32* and WRKY proteins from different species. *VbWRKY32* is boxed. The plant WRKY proteins used for the phylogenetic tree are as follows: *OsWRKY11* (AK108745), *OsWRKY24* (NC_029256), *OsWRKY30* (NP_001062148), *OsWRKY45* (AY870611), and *OsWRKY71* (NC_029257) from *Oryza sativa*; *AtWRKY2*, *AtWRKY10*, *AtWRKY11* (NP_849559), *AtWRKY15* (NP_179913.1), *AtWRKY17* (NP_565574.1), *AtWRKY18* (NP_567882), *AtWRKY20*, *AtWRKY25* (NP_180584), *AtWRKY26* (AAK28309), *AtWRKY32*, *AtWRKY34*, *AtWRKY41*, *AtWRKY53* (NP_194112), and *AtWRKY58* from *Arabidopsis thaliana*; *TaWRKY2* (EU665425), *TaWRKY19* (EU665430) from *Triticicum aestivum*; *CsWRKY2* (AFJ54352), *CsWRKY46* from *Camellia sinensis*; *VpWRKY2* (GU565706) and *VpWRKY3* from *Vitis pseudoreticulata*; *GmWRKY54* (DQ322698) from *Glycine max*; *Hv-WRKY38* from barley; *VvWRKY11* (EC935078) from *Vitis vinifera*; *BcWRKY46* (HM585284) from *Brassica campestris*; *BcWRKY46* (HM585284) from *B. campestris*.

The full-length *VbWRKY32* gene was 1,689 bp, of these, the open reading frame (ORF) was 1,500 bp and encode 499 amino acids with predicted protein molecular weight of 55.138 kDa. It contained two WRKY domains of WRKYGQK and two C2H2 zinc-finger motifs (Cx_4_Cx_22_HxH and Cx_4_Cx_23_HxH) (**Figure 1**). Comparisons of the amino acid sequences between *VbWRKY32* and other WRKY proteins in plants showed that WRKYGQK and two C2H2 zinc-finger motifs were highly conserved in four plant species (**Figure 2**). The phylogenetic tree was constructed with full-length amino acid sequences, the result demonstrated that *VbWRKY32* belonged to group I of the WRKY family (**Figure 3**).

### The Analysis of *VbWRKY32* Expression in Wild-Type Plants Under Cold Stress

**Figure 4:**
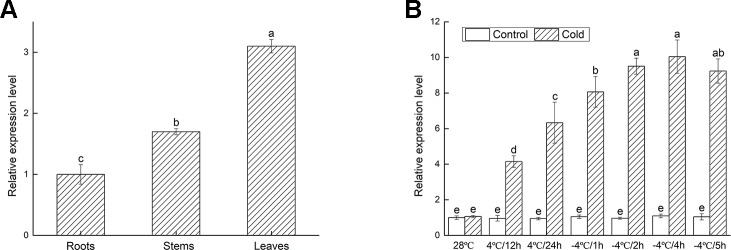
The expression analysis of *VbWRKY32* in leaves of *Verbena bonariensis*. **(A)** Relative expression level of *VbWRKY32* in roots, stems, and leaves of WT lines at 28°C. **(B)** Cold treatment. Control stands for non-stress treatment and at 28°C. The process of temperature treatment was continuous. The temperature was at 28°C, then drop to 4°C for 12 h, 24 h, and then to −4°C for 1, 2, 4, 5 h. Data indicate means ± standard errors (SE) of three biological replicates. The different letters above the columns represent significant differences (P < 0.05) on the basis of Duncan’s multiple range test.

To determine the involvement of *VbWRKY32* under cold stresses, the expression of *VbWRKY32* in different tissues of *V. bonariensis* was measured by RT-qPCR. The results were showed in **Figure 4A**. The transcript level of *VbWRKY32* was higher in leaves than that in stems and roots. The change of *VbWRKY32* expression was not significant during the course of the experiment under normal condition (28°C). The expression of *VbWRKY32* was increased during 4°C and reached a peak at 4 h during −4°C, then decreased at 5 h (**Figure 4B**). The result indicated that *VbWRKY32* was involved in the cold response.

### Overexpression of *VbWRKY32* in *Verbena Bonariensis* Enhanced the Cold Resistance

**Figure 5:**
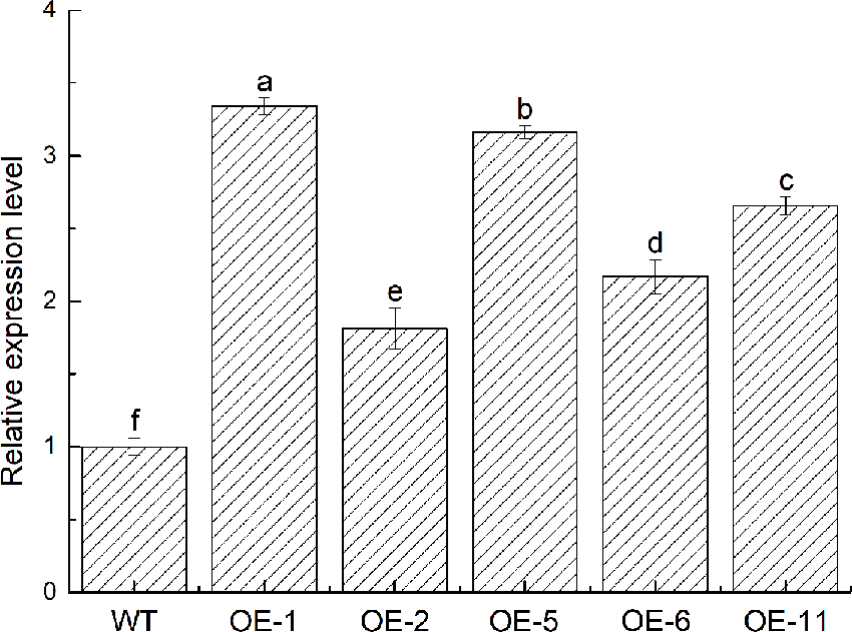
Acquisition of transgenic *VbWRKY32 Verbena bonariensis*. Expression level of *VbWRKY32* in wild-type (WT) and transgenic *V. bonariensis*. *Actin-11* served as the internal reference gene. The different letters above the columns represent significant differences (P < 0.05) on the basis of Duncans multiple range test.

**Figure 6:**
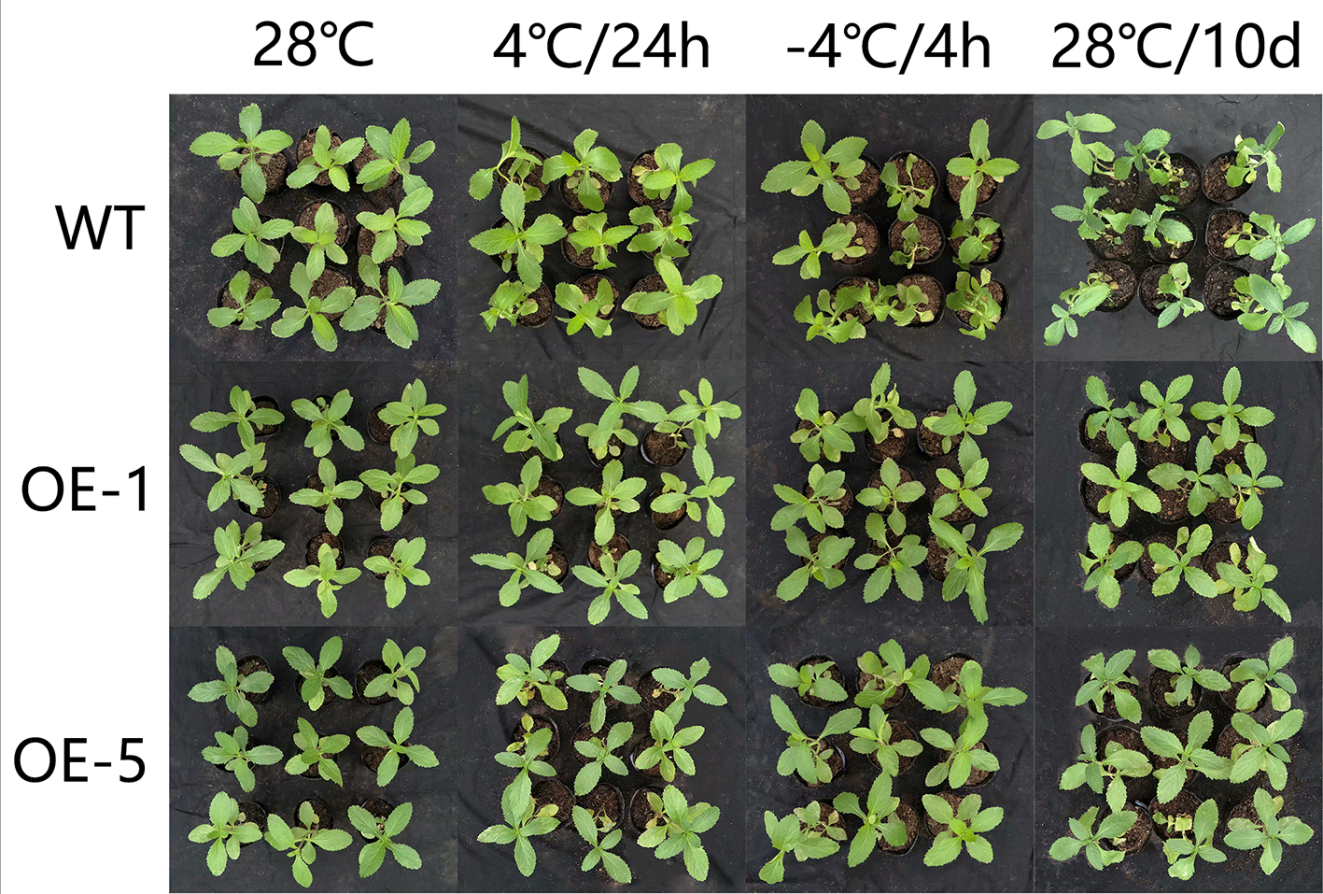
Phenotypic comparison of wild-type (WT) and *VbWRKY32* overexpressed lines (OE-1 and OE-5) under cold stress.

To further identify the function of *VbWRKY32*, *V. bonariensis* transgenic plants overexpressing *VbWRKY32* were obtained. The expression level of *VbWRKY32* in leaves was measured through RT-qPCR. The results of RT-qPCR showed the *VbWRKY32* transcript abundance of lines OE-1 and OE-5 was evidently (P < 0.05) higher than that of other OE and WT lines under 28°C (**Figure 5**). Therefore, OE-1 and OE-5 lines were selected for cold stress experiment. The plants were treated under following conditions: 4°C for 24 h, followed by −4°C for 4 h, finally allowed to recover for 10 days at 28°C (**Figure 6**). Under 28°C for 7 days, there were no obvious phenotypic difference between OE and WT lines. Under 4°C for 24 h, leaves of WT plants began to wilt, while transgenic plants’ leaves remained unchanged. The WT and OE plants were then treated at −4°C for 4 h, WT plants obviously fell over and even dead, leaves of OE lines partially wilted. The plants recovered at 28°C for 10 days, most of leaves of WT turned yellow and withered. The leaves of OE lines were not affected much and only a few of leaves turned yellow. The transgenic *V. bonariensis* showed better recovery than WT. The recovery ratio of OE-1 and OE-5 were 84.45 and 86.67%, respectively, whereas WT plants was 44.44% (**Table 3**).

**Table 3:** The statistics of seedling recovery.

	Recovery ratio	Dead ratio
WT	44.44%	4.45%
OE-1	84.45%	2.22%
OE-5	86.67%	2.22%

### The Overexpression of *VbWRKY32* Alleviated the Degree of Plants Injury

**Figure 7:**
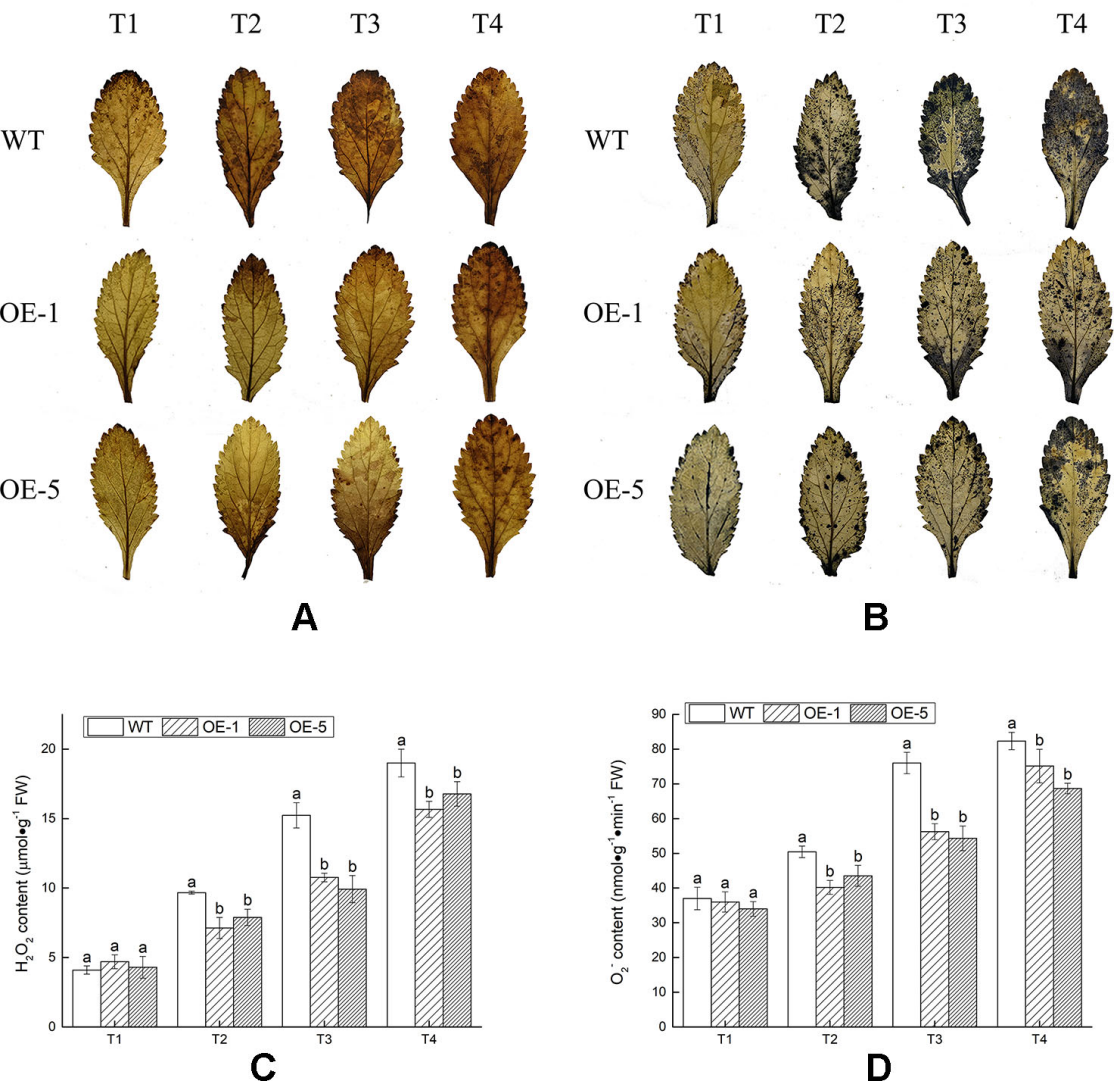
Analysis of the reactive oxygen species (ROS) accumulation levels in wild-type (WT) and *VbWRKY32* overexpression (OE)-1 and OE-5 lines of *Verbena bonariensis* (OE-1 and OE-5) under cold stress. **(A**, **B)** Histochemical staining with 3,3′-diaminobenzidine (DAB) and nitroblue tetrazolium (NBT) for observing the accumulation situation of H_2_O_2_ and O_2_^−^. **(C**, **D)** The quantitative measurement of H_2_O_2_ and O_2_^−^. The temperature kept at 28°C (T1), then drop to 4°C for 24 h (T2), then to −4°C for 4 h (T3), 6 h (T4). Data indicate means ± standard errors (SE) of three biological replicates. The different letters above the columns represent significant differences (P < 0.05) on the basis of Duncan’s multiple range test.

In the process of aerobic metabolism of plants, ROS such as H_2_O_2_ and O_2_^−^ was accumulated under cold stress, harming the membrane and related biological macromolecules. To visualize H_2_O_2_ and O_2_^−^ produced in *V. bonariensis* leaves under cold stress, the leaves were stained by using DAB and NBT chemical. Histochemical staining showed that less brown or blue precipitations were observed in overexpressed lines (OE-1 and OE-5) than that in WT (**Figures 7A**, **B**). In addition, quantitative analysis also exhibited that the accumulated level of H_2_O_2_ and O_2_^−^ in leaves of all lines were increased when exposed to cold condition, WT significantly (P < 0.05) produced more H_2_O_2_ and O_2_^−^ than OE lines (**Figures 7C**, **D**).

**Figure 8:**
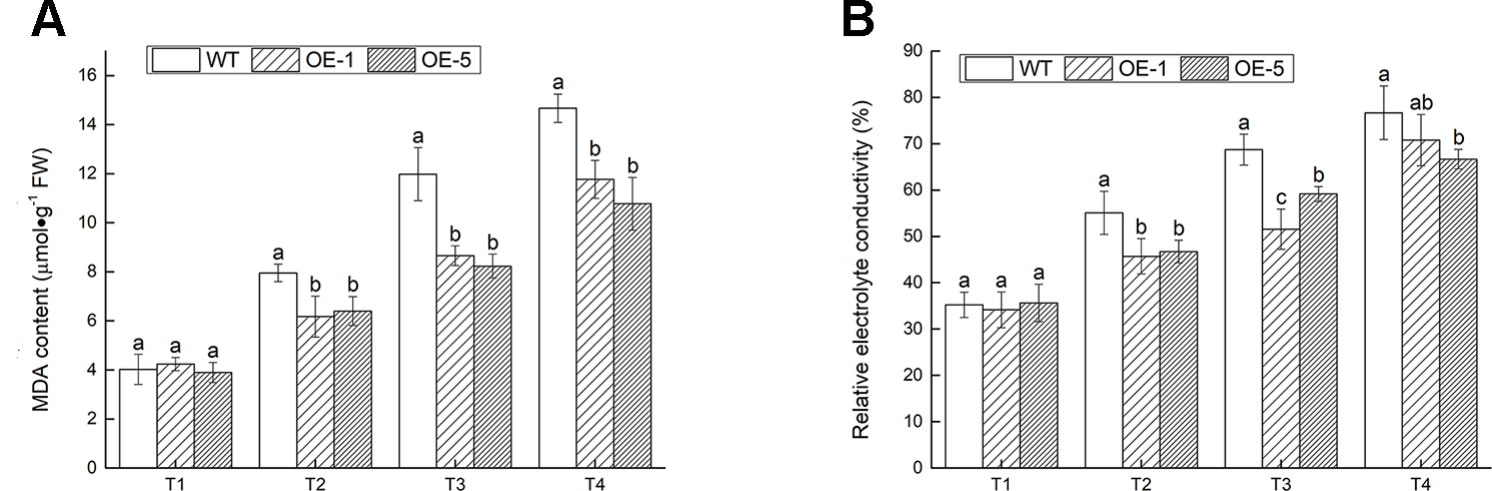
The extent of membrane injury. **(A)** Malondialdehyde (MDA) content. **(B)** Relative electrolyte conductivity. Data indicate means ± standard errors (SE) of three biological replicates. The different letters above the columns represent significant differences (P < 0.05) on the basis of Duncan’s multiple range test.

MDA and EL, the two important indexes of cell damage, could reflect the extent of membrane injury (Huang et al., 2013). The MDA content and EL were prominently (P < 0.05) lower in transgenic *V. bonariensis* than that in WT (**Figure 8**). Under T4 treatment, the MDA content of OE-1, OE-5 lines increased to 2.78- and 2.76-fold, while WT increased to 3.65-fold; The EL of WT, OE-1, and OE-5 raised to 2.18-, 2.07-, and 1.87-fold of that before stress.

The conclusion above indicated that the degree of cell damage in WT plant was more severe than the transgenic lines under cold stress.ss

### The Enhancement of Cold Tolerance by the Physiological Changes of *VbWRKY32* Overexpression

**Figure 9:**
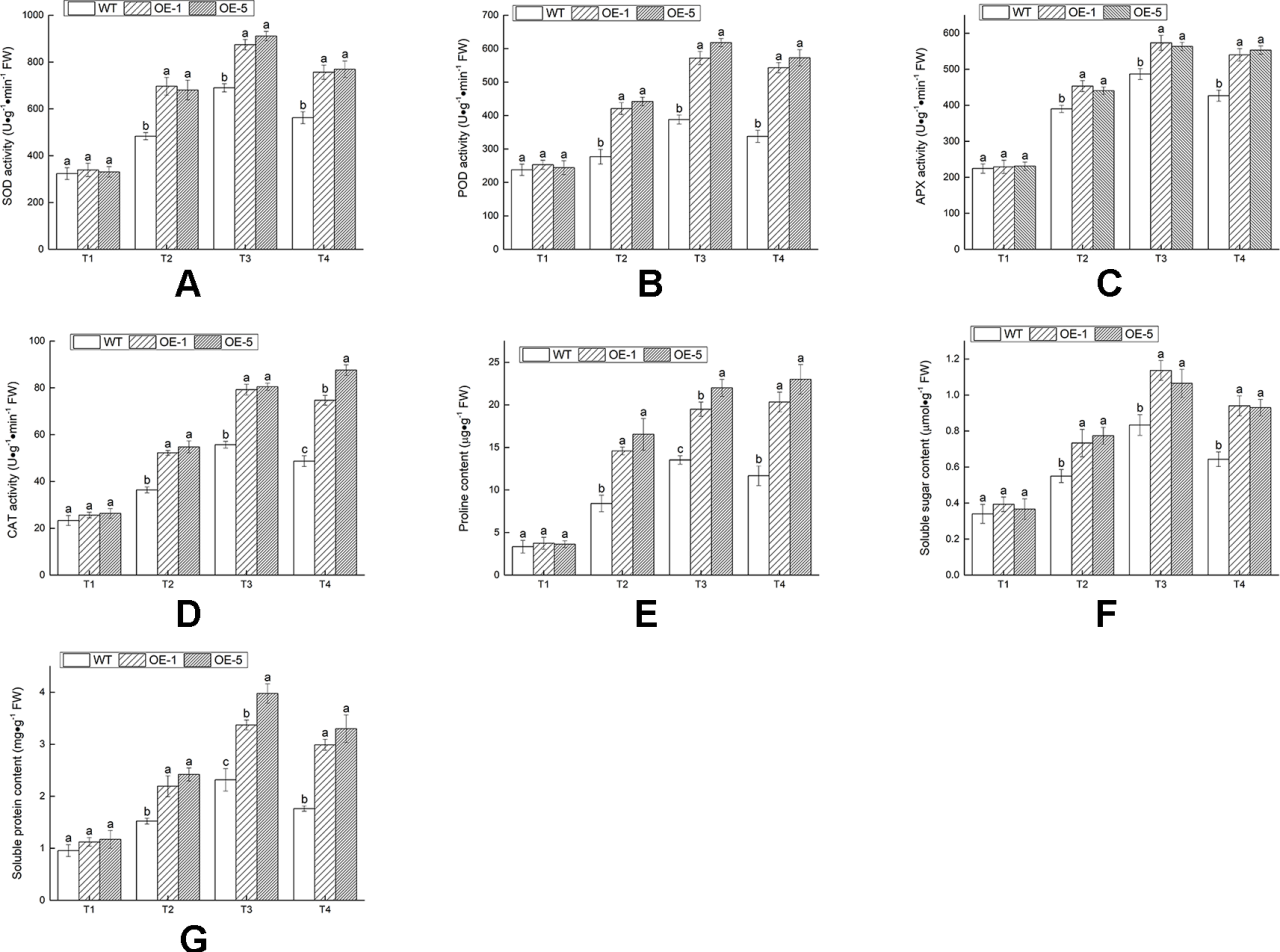
The changes of physiological indexes on wild type (WT) and *VbWRKY32* overexpression (OE) lines leaves of *Verbena bonariensis* (OE-1 and OE-5) under cold stress. **(A)** Superoxide dismutase (SOD) activity. **(B)** Peroxidase (POD) activity. **(C)** Ascorbate peroxidase (APX) activity. **(D)** Catalase (CAT) activity. **(E)** Proline content. **(F)** Soluble sugar content. **(G)** Soluble protein content. Data indicate means ± standard errors (SE) of three biological replicates. The different letters above the columns represent significant differences (P < 0.05) on the basis of Duncan’s multiple range test.

The existence of antioxidant enzymes was essential for scavenging ROS and alleviating cell injury. Hence the activities of antioxidant enzymes (SOD, POD, APX, and CAT) at various time and temperature points were monitored (**Figures 9A**–**D**). Under 28°C condition (T1), these enzymes activities revealed no remarkable difference in between WT and OE lines. After chilling treatment of −4°C for 4 h (T3), there was an evident increase in all lines; exposure to −4°C treatment for 6 h (T4), the activities of SOD, POD, and APX decreased in OE and WT lines. The contents of proline, soluble sugar, and soluble protein were measured to investigate the regulated capable of osmotic mechanism in *VbWRKY32* OE lines of *V. bonariensis* under cold stress. Compared with WT, OE lines accumulated distinctly (P < 0.05) higher content of proline, soluble sugar, and soluble protein under cold condition (**Figures 9E–G**). In addition, SOD, POD, APX, and CAT were normalized by protein data (**Figure S1**). As a whole, the value of SOD, POD, APX, and CAT after normalized treatment were lowered in OE lines than that in WT during cold condition.

These results indicated that overexpression of *VbWRKY32* could raise the ability of plants against ROS persecution and alleviate cell injury under cold stress.

### Differential Expression of Cold-Related Genes in Wild-Type and Overexpression Lines Under Cold Stress

Based on the above physiological data of *V. bonariensis* under low temperature stress, it was found that the antioxidant enzyme activity and osmotic adjustment substance content of plants reached a high level under −4°C for 4 h. Considering that the physiological changes depended on the change of molecular mechanism, it was speculated that the gene expression should be at a high level at this time. Therefore, in order to understand the effect of *VbWRKY32* overexpression on the cold resistance at gene level, expressions of nine cold-related genes were detected by qRT-PCR at T1 (28°C), T2 (4°C), and T3 (−4°C).

**Figure 10:**
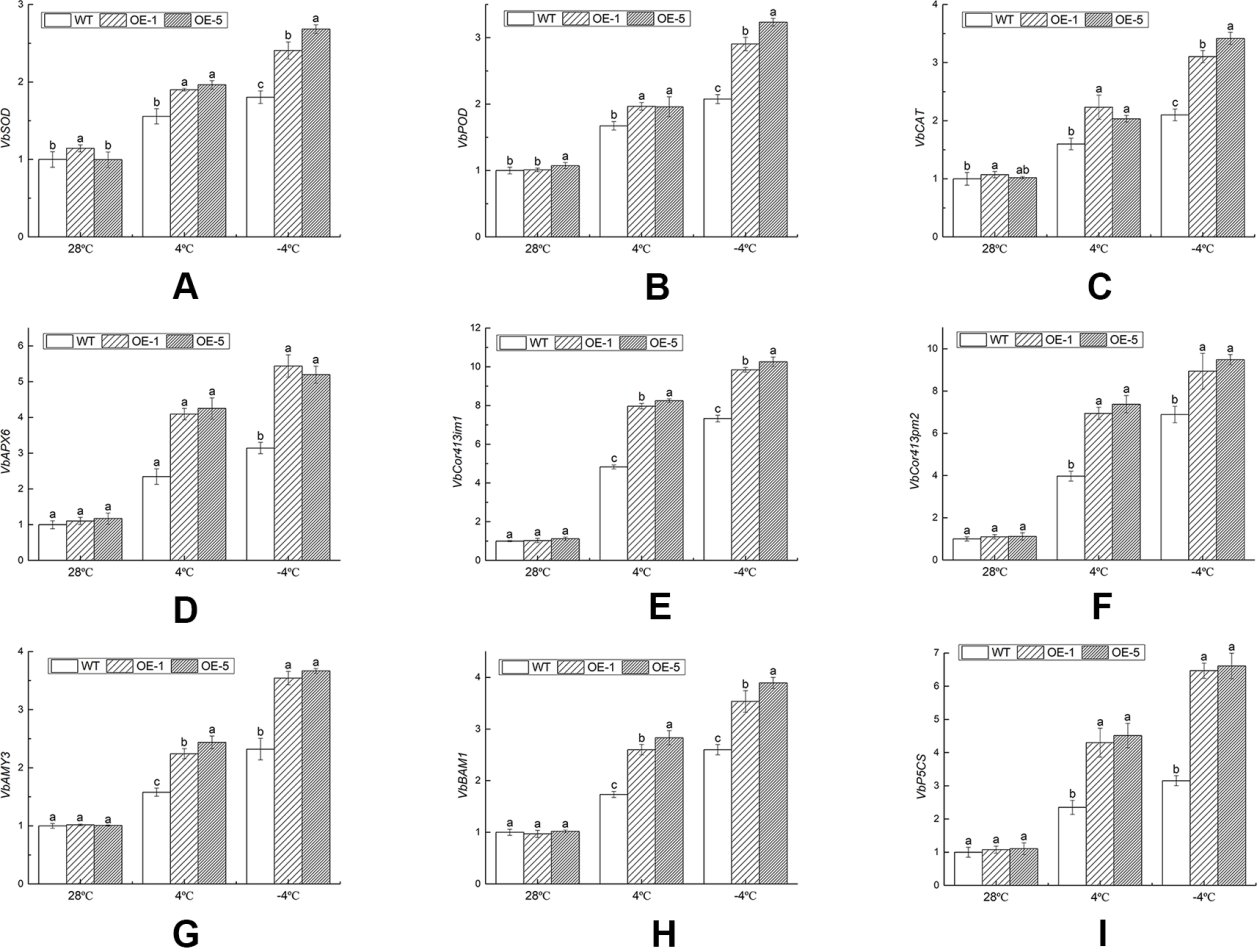
The expression analysis of transcripts of stress-response genes in wild-type (WT) and overexpression (OE) lines (OE-1 and OE-5) at various time and temperature points. **(A)** *VbSOD*. **(B)** *VbPOD*. **(C)** *VbCAT*. **(D)** *VbAPX6*. **(E)** *VbCor413im1*. **(F)** *VbCor413pm2*. **(G)** *VbAMY3*. **(H)** *VbBMY1*. **(G)** *VbP5CS*. *Actin-11* was amplified as a control. Data indicate means ± standard errors (SE) of three biological replicates. The different letters above the columns represent significant differences (P < 0.05) on the basis of Duncan’s multiple range test. **(I)** The seedlings were treated at the following temperature (this cooling process was continuous.): 28°C (the control), 4°C for 24 h, followed by −4°C for 4 h.

These genes selected were related to antioxidant enzyme, cold regulated protein, and osmotic adjustment substances. The antioxidant enzyme activities and the content of osmotic adjustment substances were increased, which helped clear excess ROS, maintain the balance of cell osmotic pressure, and stabilize the cell structure. Under normal environment, there was no remarkable difference in transcript accumulation between WT and OE lines. Under chilling stress (4°C), the gene expression levels of OE lines were specifically up-regulated than that of WT. Compared with the control (28°C), *VbSOD* in the WT, OE-1, and OE-5 lines was remarkably (P < 0.05) increased by 1.80, 2.10, and 2.69 times under freezing stress (−4°C) (**Figure 10A**), respectively. The gene expression level of *VbPOD* and *VbCAT* matched similar pattern with *VbSOD*, which was 3.01 and 3.35 times in OE-5 lines, respectively, higher than that of the control (**Figures 10B**, **C**). *VbAPX6* in the WT, OE-1, and OE-5 lines was increased to 3.14, 4.93, and 4.44 times under −4°C than that of the control (**Figure 10D**). The transcription levels of *VbCor413im1* in OE lines were over 7.56-fold at 4°C and 9.13-fold at −4°C, greater than that in control, respectively; the expression level of *VbCor413pm2* in OE lines was more than 6.30 times at 4°C and 8.11 times at −4°C higher than that of WT, respectively (**Figures 10E**, **F**). The expression level of *VbAMY3*, *VbBAM1*, and *VbP5CS* were signiﬁcantly (P < 0.05) increased by over 3.49, 3.64, and 5.99 times in OE lines under −4°C stress than that of control, a maximum reached (**Figures 10G**–**I**). The results indicated that *VbWRKY32* transcription factor could increase the expression level of cold-related genes and the cold tolerance of transgenic plants.

## Discussion

Despite low-cost cultivation and high-ornamental value, the study on *V. bonariensis* has generally been slower compared with many landscape plants, especially in terms of the abiotic stress response. Cold stress decrease production and ornamental value of *V. bonariensis*. The WRKY TF family was paid great attention to in the field of abiotic stress. It represented as one of major plant-specific transcriptional regulators. Many WRKY TFs were up-regulated under drought, heat, or salt stresses (Wang et al., 2018b; Xu et al., 2018). However, few researches reported the function of WRKY TF under cold stress. In our experiment, *VbWRKY32* was separated from *V. bonariensis* on its differential expression in response to freezing stress.

The results of multiple sequence alignment and phylogenetic tree analyses showed that the *VbWRKY32* gene belonged to the group I of WRKY family. The transgenic plants of *VpWRKY2*, which are in the same branch as *VbWRKY32*, possessed high cold resistance (Li et al., 2010). Overexpression of *TaWRKY19* enhanced the ability of responding salt, drought, and freezing stresses in transgenic plants (Niu et al., 2012). In addition, *WRKY32* was actively upregulated in kenaf responding to drought and salinity stresses (Niu et al., 2015). Moreover, the expression level of *VbWRKY32* in leaves was remarkably raised under cold stress. The above results showed that *VbWRKY32* could participate in the cold resistance process in *V. bonariensis*.

The expression level of *VbWRKY32* in leaves is higher than in stems and roots and the transcript peak was after 4 h at −4°C. The leaves reflected the chilling and freezing injury. We speculated that the leaves activated the expression of *VbWRKY32* more quickly than other tissues exposed directly to cold air. CsWRKY2 expression was highly promoted in leaves than in other organs exposed to coldness, drought, and exogenous abscisic acid (Wang et al., 2016). The previous result may be consistent with our speculation. The *VbWRKY32* gene in WT could increase expression under cold stress. The elevated expression of *VbWRKY32* transgenic lines under normal circumstances could account for its rapid response to cold. The damage of WT plants could have occurred in the early stages responding to cold stress in the process of resistance.

In the experiment for exploring the cold tolerance of *V. bonariensis*, the OE lines were promoted in response to chill compared with WT. The results were revealed by observing phenotypic changes and measuring recovery ratios, MDA, EL, antioxidant enzyme activities, and osmotic regulating substance contents. *VbWRKY32* overexpression caused the elevated expression of down-stream genes and the changes of their related substances in plants.

The MDA content and EL could be used to test the degree of lipid peroxidation and the change of membrane permeability, respectively. Due to cold stress, the high concentration of ROS which was reactive and toxic caused the enhanced production of MDA. The accumulation of MDA under abiotic stress, which caused membrane lipid peroxidation, injury of plant cells, lead to the death of plants ultimately (Mittler et al., 2004; Wang et al., 2018a). Under the chilly condition, the cell membrane of plant transformed from liquid crystalline phase to gel phase with the deviation of selective permeability, which resulted in the cellular electrolyte exosmosis. Therefore, the damage of plants under low temperature stress could be measured by MDA and EL. The production of MDA and the EL of WT were greater compared with *VbWRKY32* OE lines. The results illustrated that *VbWRKY32* might reduce the accumulation of MDA and stable cell membrane structure.

The antioxidant enzyme system, including POD, SOD, CAT, and APX, could scavenge excessive ROS and improve plant resistance under various types of abiotic stresses (Yu et al., 2014; Singh et al., 2017; Xu et al., 2017; Ma et al., 2018). Compared with the OE lines, the WT plants showed deeper intense histochemical staining by cold treatment. The appearance suggested that less ROS was accumulated in OE lines than the WT. To scavenge ROS, the expression level of ROS-scavenging related genes (e.g., *VbSOD*, *VbPOD*, *VbCAT*, and *VbAPX6*) had a significant increase in OE lines than that in WT. The transcript levels of these genes were in line with the antioxidant enzyme (SOD, POD, CAT, and APX) activities. The antioxidant enzymes converted toxic superoxide radicals to harmless ion and eliminated hydrogen peroxide in plants (Sarvajeet Singh and Narendra, 2010). The activities of SOD, POD, CAT, and APX in OE lines were remarkably (P < 0.05) increased than that in WT. However, intrinsic antioxidant systems of plants only eliminate a certain amount of ROS and excessive ROS could destroy antioxidant systems.

During cold treatment, we found the enzymes activities of SOD, POD, APX, and CAT were higher in OE lines than that in WT. On the one hand, this may be due to an increase in the level of transcription, which in turn leaded to an increase in protein activities. Further studies need to determine the relation of the transcription of SOD, POD, APX, and CAT to protein activities. On the other hand, the protein normalization treatment of SOD, POD, APX, and CAT was performed, the results have showed that the concentration of enzymes (including protein per FW) in OE lines were lower than that in WT (**Figure S1**). While the decreases in MDA, H_2_O_2_, O_2_^−^ content (expressed per g FW) in the WRKY OE lines might be explained by an increased retention of water in the OE lines. However, SOD, POD, APX, and CAT (expressed per g FW) in the OE lines increased and they are no significantly different when normalized for protein (expressed per mg protein, **Figure S1**), it was speculated that increased water loss of in OE lines. These two phenomena were contradictory and not easy to explain. Therefore, the relation of the loss water of plants under cold stress to the protein activities needed further study.

The osmotic adjustment substances such as soluble sugar, soluble protein, and free proline could keep the stability of cellular structure and cell osmotic pressure. It was proved that they aided to maintain proteins and cell structures, particularly under severe or prolonged stress (Hoekstra et al., 2001). Under freezing stress, the transcription levels of *VbAMY3*, *VbBAM1*, and *VbP5CS* had been greatly improved, the expression levels of these gene in OE lines were evidently higher than WT. The expression levels of *AMY3* increased significantly under cold conditions in *Arabidopsis* (Thalmann et al., 2016). By degrading starch into maltose under stress, *BAM1* sustained the biosynthesis of proline and soluble sugars, alleviating the oxidative stress (Kyonoshin et al., 2009). The lack of *AMY3* and *BAM1* prevented plants from mobilizing starch in leaves in face of stress. Carbon exported to the root was reduced, which ultimately affected osmolyte accumulation for water, nutrient intake, and root growth (Zanella et al., 2016). The expression level of *VbP5CS* which functioned in osmotic adjustment was up-regulated in OE lines. The expression of the *P5CS* gene induced by environmental stress could promote proline synthesis and increase proline content in plants (He et al., 2018). Therefore, the proline, soluble protein, and soluble sugar in OE lines was of improved content compared with WT under freezing stress.

Massive studies had revealed that expression of COR genes was positively related to cold tolerance in plants (Liang et al., 2011; Wathugala et al., 2011; Wan et al., 2014). The Cor413 family were divided into Cor413-inner membrane (Cor413im) and Cor413-plasma membrane (Cor413pm) proteins (Ghislain et al., 2003). In this study, the expression level of *VbCor413im1* and *VbCor413pm2* were up-regulated in OE lines compared with WT. *Arabidopsis AtCor413im* mapped in the inner membrane of chloroplasts might stabilize the chloroplast membrane under cold stress (Okawa et al., 2010; Okawa et al., 2014). Overexpression of *PsCor413im1* isolated from *Phlox subulata* improved cold resistance of *Arabidopsis* plants (Zhou et al., 2018b). *PsCor413pm2*, a plasma membrane protein, enhanced cold tolerance of transgenic *Arabidopsis*. The plasma membrane suffered most from cold injury (Zhou et al., 2018a).

The TFs played an important role in activating multiple biological processes to insulate plant cells from cold. They functioned as a pivotal regulator for adaption of the plant through the binding of TFs to cognate cis-acting elements present in the promoter region of their target genes (Zou et al., 2010; Yang et al., 2012; Peng et al., 2013). The downstream-related genes were regulated by TFs to adapt to external environmental changes. The cold tolerance in plants was enhanced by regulating various series of cold-responsive genes (Thomashow, 1999).

Therefore, the data indicated *VbWRKY32* improve the activities of antioxidant enzymes and the content of osmotic adjustment substances by influencing downstream genes, and then alleviate the oxidative damage and stabilize the plasma membrane of *V. bonariensis* under cold stress.

It is worth mentioning that in addition to affecting the expression of downstream genes and physiological changes, WRKY may also have an impact on water retention, and thus increase the cold resistance of plants. In *Arabidopsis*, the *WRKY54*-*WRKY70* double mutants exhibited clearly enhanced tolerance to osmotic stress. The enhanced tolerance was correlated with improved water retention and enhanced stomatal closure (Li et al., 2013). The water retention also may be related to EL, enzyme activities, MDA, H_2_O_2_, O_2_^−^ and proline. In addition, the *VbWRKY32* over-expression transgenic plants were validated by qRT-PCR in our study, the *VbWRKY32* gene played a vital role in improving cold stress tolerance. Whether there will be changes in protein levels in the transgenic plants in cold stress, further studies need to determine the relation of the VbWRKY32 gene to protein level. These issues will be focused in the future.

## Conclusion

Taken together, the overexpression of *VbWRKY32* in the seedling stage of *V. bonariensis* resulted in elevated cold tolerance without abnormal growth by phenotypic observation. The expression level of down-stream genes was remarkably promoted, which accounted for the improvements in antioxidant enzyme activities and the contents of osmotic adjustment substance. The *VbWRKY32* gene alleviated the damage of membrane lipid peroxidation and relieved the electrolyte exosmosis of the cell. These performances proved the positive function of *VbWRKY32* in *V. bonariensis* under cold stress. Currently, this cold resistance experiment was provisionally limited to the laboratory. Further exploration and verification would be required on whether the transgenic *V. bonariensis* plants’ safely survival and winter duration in the field environment below 4°C.

## Data Availability Statement

The raw sequencing data of the RNASEQ experiment have been submitted to the NCBI Sequence Read Archive database with accession number GSE112477.

## Author Contributions

M-qW, YL, and Q-lL conceived and designed the experiments. M-qW, Q-xH, PL, and Q-hZ performed the experiments. M-qW, LZ, Y-zP, B-bJ, and FZ analyzed the data. M-qW wrote the paper. All authors read and approved the final manuscript.

## Funding

This research was supported by National Natural Science Foundation of China (31971707), Sichuan Science and Technology Program (2019YJ0512), Natural Science Foundation of Guizhou (20181044), the introduction of talent project of Guizhou University (201756), and Guizhou University cultivation project (20175788-33).

## Conflict of Interest

The authors declare that the research was conducted in the absence of any commercial or financial relationships that could be construed as a potential conflict of interest.

## Supplementary Material

Figure S1 The protein normalization treatment of SOD, POD, APX and CAT. **(A)** SOD. **(B)** POD. **(C)** APX. **(D)** CAT.
