# Supplementary figures and images for: The Overexpression of a Transcription Factor Gene VbWRKY32 Enhances the Cold Tolerance in Verbena bonariensis
Source: Front Plant Sci. 2020 Jan 29;10:1746. doi: 10.3389/fpls.2019.01746 (PMC7000379; doi:10.3389/fpls.2019.01746)

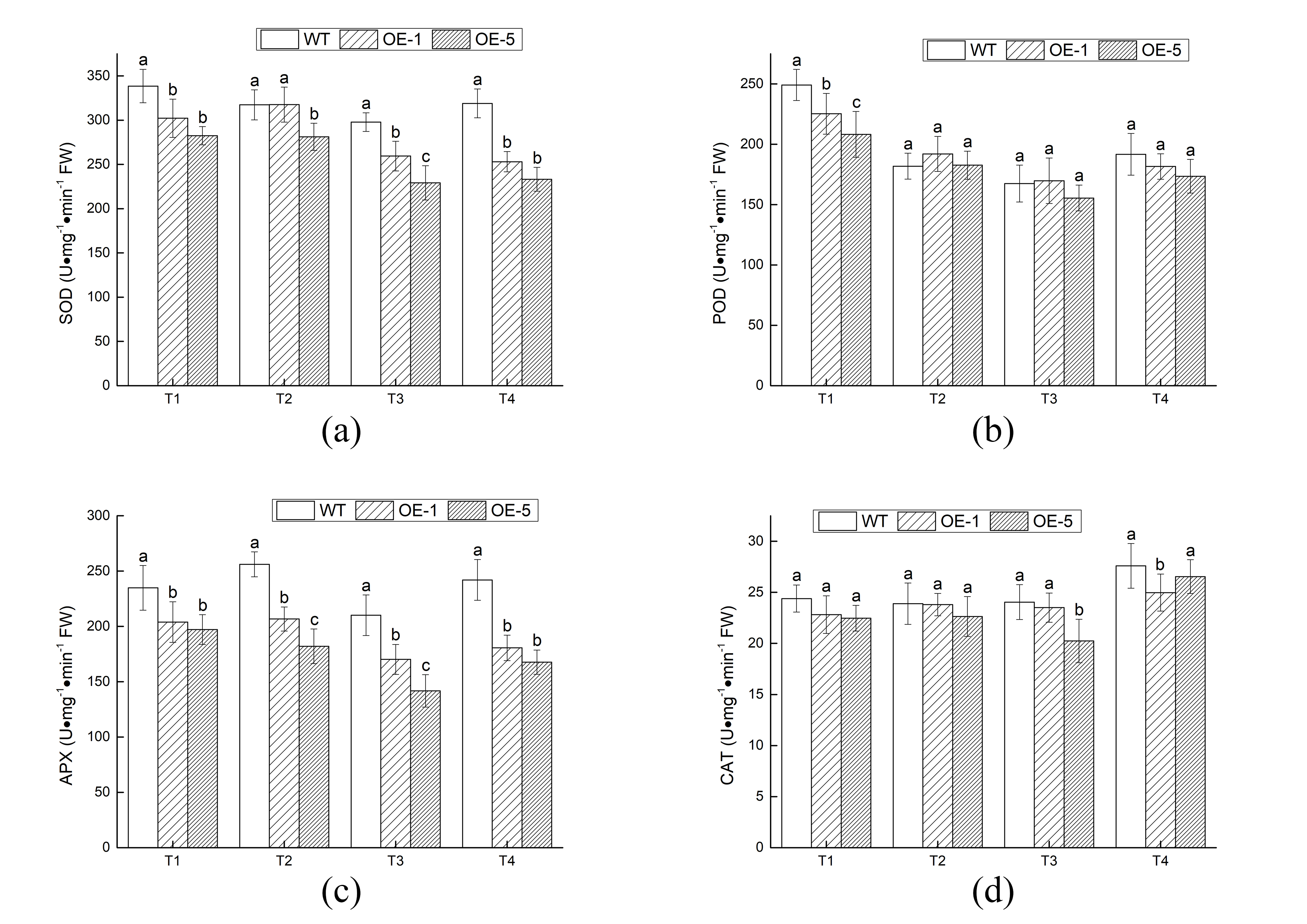

Supplement: Figure S1 — The protein normalization treatment of SOD, POD, APX and CAT. (A) SOD. (B) POD. (C) APX. (D) CAT. [file Image_1.tif]
